# End‐to‐end film‐based characterization of gated delivery latencies in a spot‐scanned synchrotron‐based proton therapy system

**DOI:** 10.1002/acm2.70826

**Published:** 2026-10-21

**Authors:** Z. S. Stottler, J. J. Kruse, A. J. Deisher

**Affiliations:** ^1^ Department of Radiation Oncology Mayo Clinic Rochester Minnesota USA

**Keywords:** gating, latency, probeat, proton, RPM

## Abstract

**Background:**

Temporal mismatches between gating signals and beam activation—beam‐on and beam‐off latencies—in gated particle beam therapy can compromise dose integrity. Yet standardized measurement techniques are lacking, and system‐specific characterization, particularly for synchrotron‐based proton systems, remains limited.

**Purpose:**

To implement a novel strategy for precise end‐to‐end gating latency measurements of a spot‐scanned proton beam using Gafchromic film.

**Methods:**

Gating latencies have been measured for a Hitachi ProBeat accelerator equipped with a Varian RPM gating system. EBT4 films were positioned within a stack of acrylic blocks on a QUASAR respiratory motion platform operated with a 1.5 cm amplitude and 12 bpm sinusoidal pattern. A stainless‐steel ball bearing (BB), positioned atop the film, facilitated localization. During irradiation, the BB produced a dose shadow on the film that indicated the phantom position. A proton plan consisting of a single 85.3 MeV spot was delivered to isocenter during a single gate‐on/off interval initiated near the 25% phase, to maximize phantom velocity. Fifty‐one measurements were made with a variety of gating intervals. Beam‐on latency and delivery time produced a separation between the BB‐shadow and the centroid of the Gaussian proton spot. Spot duration information was extracted from accelerator log files. BB‐shadow/spot separation is modeled analytically based on phantom motion enabling the direct calculation of the total beam‐on and beam‐off latencies. Accelerator latency was measured directly with an oscilloscope, enabling isolation of the relative contributions of the RPM gating system.

**Results:**

An average beam‐on latency of 142±27 ms is observed for the RPM+ProBeat system for the first gate of a delivery, which includes a known 69 ms beam extraction judgment time for the first proton spot in a spill from the Hitachi synchrotron. The corresponding average beam‐off latency is 87±30ms. The average gate‐on and gate‐off latencies of the RPM system are calculated as 72±27ms and 81±30ms, respectively. The associated average contributions of the accelerator to the beam‐on latencies are measured as 69.3±0.2ms (first gate in new spill) and 0.53±0.07ms (subsequent gates in existing spill) and similarly 6±4ms for beam‐off latency.

**Conclusion:**

A novel, high‐precision method for gating‐latency measurements has been implemented using film to characterize gating latencies in a spot‐scanned particle beam. Commissioning and regular quality assurance methods to precisely characterize the temporal accuracy of a gating system ensure treatment accuracy and will become increasingly vital to consider for higher‐frequency applications, such as cardiac gating or FLASH‐capable systems.

## 1 INTRODUCTION

In scanned particle beam therapy, respiratory motion presents a significant challenge for targets located in the thorax and abdomen, where internal target motion due to respiration may compromise the integrity of the delivered dose distribution.^1^ Interplay between internal motion and the time‐dependent nature of the scanned particle beam may spoil target coverage, increase dose to normal tissues, and in some cases produce unacceptably large dose heterogeneity.^2^, ^3^, ^4^ The American Association of Physicists in Medicine (AAPM) Task Group (TG) 290^1^ describes the use of passive strategies—including motion‐robust beam‐angle selection, decreased spot spacing, and rescanning/repainting techniques—and active motion management techniques—such as respiratory phase‐gating and breath holding—to mitigate motion‐induced range uncertainties and interplay effects. Respiratory Phase‐Gating (PG) synchronizes beam delivery with a predefined portion of the breathing cycle.^5^, ^6^ PG systems correlate phases of the respiratory cycle with the motion of an externally positioned surrogate. At simulation, external surrogate displacements are correlated with internal target motion and respiratory phase. A subset of phases, called the gating interval, is then identified to restrict target motion to a tolerable level during treatment delivery, thereby minimizing motion‐related uncertainties. During treatment, PG systems permit beam delivery during the specified gating interval. However, the implementation of accurate PG is a complex challenge, as it relies on the precise temporal alignment of surrogate position with beam activation. Any temporal mismatches—in this work labeled beam‐on or beam‐off latency—can result in unintended dose delivery, possibly leading to a geographic miss or unintended irradiation of healthy tissues.

AAPM TG‐142^7^ has established a recommendation, in the context of gated x‐ray therapy, that the temporal accuracy of respiratory gating systems not exceed 100ms. This level of temporal accuracy ensures that delays do not induce a positional error exceeding 2mm, provided target motion does not exceed 2cm/s. While prevention of a geometric error is one concern in gated particle‐beam therapy, the mitigation of patient‐ and delivery‐specific motion‐induced interplay effects must necessarily be taken into account.^1^ TG‐142 established the first quantitative benchmark for gating‐system temporal accuracy in 2009; yet, many commercially available gating‐systems struggle to satisfy this requirement, particularly when compensating for irregular or complex respiratory motion patterns.^8^, ^9^, ^10^ Moreover, latency characterization is complicated by the lack of a standardized measurement technique, with existing vendor solutions providing only indirect estimates that may not capture the full range of system delays. Despite the risks posed by unconstrained and uncharacterized latencies in gated particle beam deliveries, the recently published AAPM TG‐290 makes no mention of latency, nor does it advance any recommendations on how latency may be characterized.

The aim of this study is to implement a film‐based method for precise end‐to‐end characterization of gating latencies. Measurements were performed for a Hitachi ProBeat synchrotron equipped with the Varian Real‐time Position Management (RPM) system. Concurrent monitoring of the gate and beam‐extraction signals with an oscilloscope permits the decomposition of total latencies into their gating‐system and accelerator components. This work establishes a practical methodology for commissioning and quality assurance of gated proton beam delivery delays. To the authors' knowledge, this constitutes the first complete characterization of the total and constituent gating latencies for the RPM + Hitachi ProBeat system.

## 2 METHODS

### 2.1 Proton facility and gating system

Gated delivery latencies have been measured for a clinical Hitachi ProBeat spot‐scanning proton therapy system (Hitachi America, Ltd., Santa Clara, CA, USA) equipped with the Varian Real‐time Position Management (RPM) system (Varian Medical Systems, Palo Alto, CA, USA), version 1.7. All measurements were made for a single gantry in a multiroom facility. Unlike a cyclotron facility, a synchrotron delivers beam in discrete spills with associated beam extraction judgment times. The proton beam is gated on/off by RPM via a 5‐volt gating signal passing through a custom interface that provides compatibility between the RPM and Hitachi systems.^6^ The (monoscopic) RPM system employs a couch‐mounted infrared (IR) camera to track a pair of IR‐reflective markers affixed to a lightweight plastic block, which is typically positioned on a patient's chest or abdomen during treatment. The RPM camera operates with a frame rate of 30 Hz; acquired images are analyzed to reconstruct the motion of the marker block, which is correlated with the patient's respiratory motion. The RPM software employs a proprietary predictive filter to monitor and forecast the patient's breathing pattern. Due to its proprietary nature, the exact details of the underlying algorithm are unknown. RPM has a user‐specified regularity enforcement parameter (accepted value range: 0–20), which determines the degree to which variability in patient breathing is tolerated. To match our clinical practice, the regularity enforcement parameter was set to a value of zero.

### 2.2 Experimental setup

For each experiment, a $4\times 4\;{\text{cm}}^{2}$ piece of EBT4 film (Ashland, Wilmington, DE, USA) was placed within a stack of acrylic blocks set on a horizontal motion platform (QUASAR Respiratory Motion Platform, Modus QA, Ontario, Canada). The motion platform was driven with a 1.5 cm amplitude (3 cm peak‐to‐trough) cosine waveform at a realistic respiratory rate of 12 cycles per minute, corresponding to a period of 5 s. The RPM marker block, henceforth called the surrogate, was placed on the vertical motion stage that is mechanically synchronized with the horizontal respiratory motion platform.

A stainless‐steel ball bearing (BB) embedded within the acrylic slab placed atop the film facilitated localization and film marking. Figure 1 shows this experimental setup. In preparation for each measurement, the phantom was driven to the alignment phase $\varphi_{a}=25\%$ and the BB was radiographically aligned to isocenter; phantom motion was subsequently initiated. During irradiation, the BB produced a shadow in the dose deposited on the film, a technique we employ in‐house for routine QA of the coincidence of imaging, mechanical, and radiation isocenters.^11^ For each measurement, a single 85.3 MeV proton spot was delivered to isocenter during a single gate‐on interval. The gantry was positioned at $0^{\circ }$ such that the beam was normally incident on the surface of the film. The beam energy was selected to optimize the relative fraction of protons that stop in the BB, thereby maximizing the contrast of the BB‐shadow. Once in motion, the phantom velocity attains a maximal value of approximately 18.8mm/s at the 25% phase. During irradiation, the film moved orthogonally with respect to the beam, smearing out the approximately Gaussian dose distribution. Nonzero beam‐on latency increased the separation between the BB‐shadow and the centroid of the Gaussian dose distribution recorded on the film.

**FIGURE 1:**
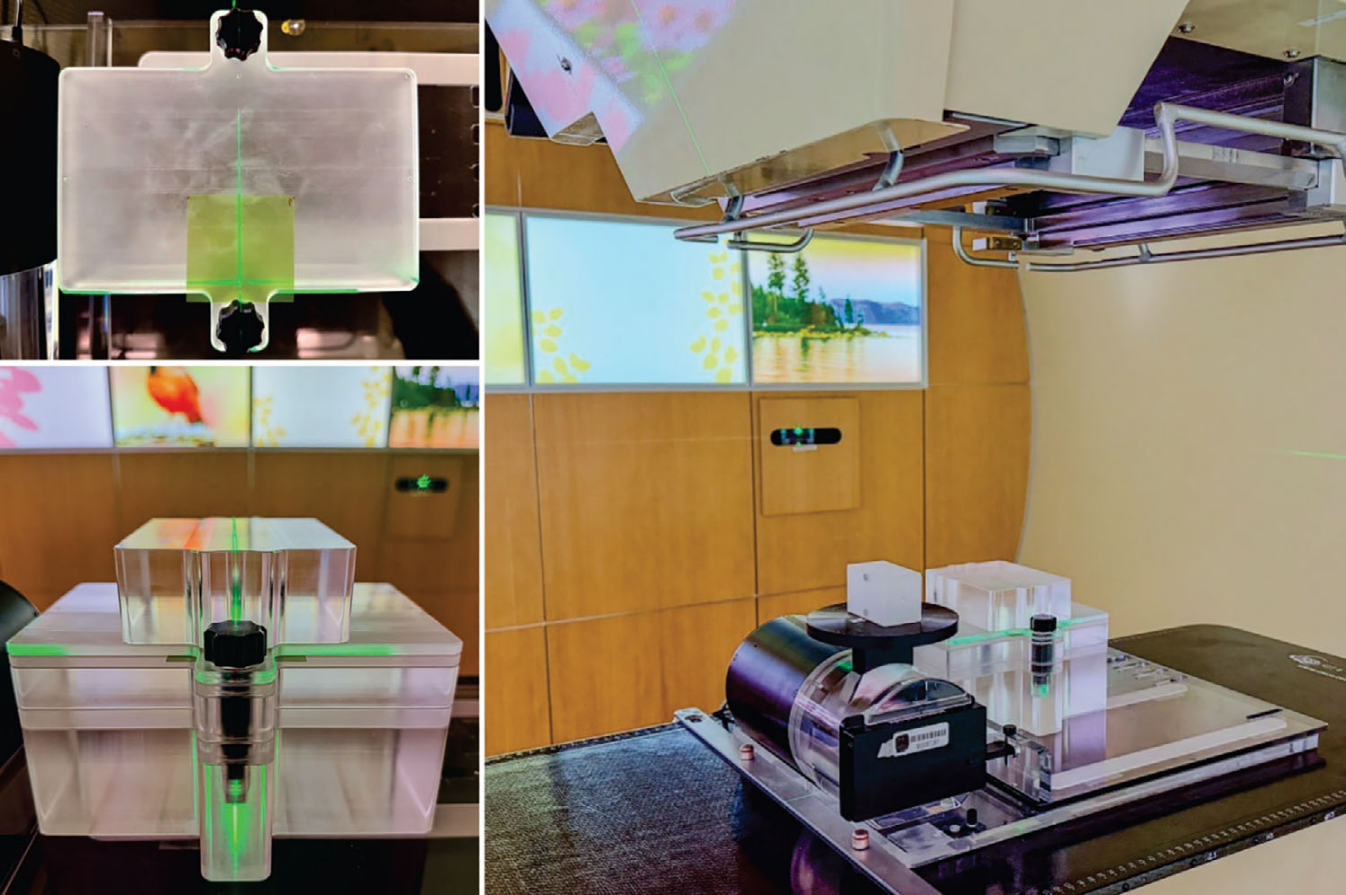
Experimental setup: a piece of EBT4 film positioned beneath a steel BB in a (custom) acrylic phantom on a QUASAR respiratory motion platform, which has been radiographically aligned to isocenter (indicated by the green lasers). Right: setup overview. Upper left: top‐down view with the upper acrylic block removed. Lower left: side view with the film in place.

Films were scanned with a high spatial resolution (200 pixels per inch) at least 24 h after measurement to maximize the measurable signal.^12^ All films were scanned individually by positioning them near the center of an EPSON 13000XL flatbed scanner; analysis was performed in Film QA Pro (Ashland, Wilmington, DE, USA). The clinical calibration curve for the relevant batch of EBT4 film was used to convert the reading to dose. The under‐response of gafchromic film in regions of high Linear Energy Transfer (LET) has been well documented.^13^ In the context of this work, the position, not the magnitude, of the dose on the film was important, so LET corrections have been neglected. Where relevant, we report dose in arbitrary units (a.u.). BB‐shadow/spot separation was extracted with a two‐dimensional (2D) fit to the dose distribution using the RooFit package^14^ available within ROOT.^15^

During each experiment, a Tektronix TDS2004C oscilloscope (Tektronix, Beaverton, OR, USA), operated with a 2.5 kHz sampling rate (corresponding to 0.4 ms/sample), was used to monitor the status of the gate‐on/off and beam‐on/off signals. The surrogate for beam‐on/off was provided by a 5‐volt (binary) logic signal, accessible within the control room, which indicates the state of the kicker magnet responsible for beam extraction from the Hitachi synchrotron. The timing relationships among the surrogate, gate, and beam signals are illustrated schematically in Figure 2. The spot's delivered position with respect to isocenter and its timing information were extracted from accelerator log files.

**FIGURE 2:**
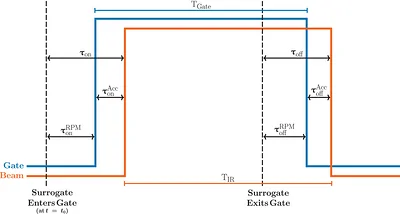
Timeline of signals in a gated delivery; the surrogate position is indicated by the vertical dashed black lines, the solid blue line shows the gate‐on/off signal, and the solid orange line shows the accelerator beam‐on/off signal.

We define the total beam‐on latency $\tau_{\text{on}}$ as the sum of the RPM gate‐on latency $\tau_{\text{on}}^{\text{RPM}}$ and the accelerator beam‐on latency $\tau_{\mathrm{on}}^{\text{Acc}}$

(1) $$\tau_{\text{on}}=\tau_{\text{on}}^{\text{RPM}}+\tau_{\text{on}}^{\text{Acc}}.$$

Similarly, the total beam‐off latency $\tau_{\text{off}}$ is defined as the sum of the respective components for the gate‐off $\tau_{\text{off}}^{\text{RPM}}$ and accelerator beam‐off $\tau_{\text{off}}^{\text{Acc}}$ latencies

(2) $$\tau_{\text{off}}=\tau_{\text{off}}^{\text{RPM}}+\tau_{\text{off}}^{\text{Acc}}.$$

We define the time that the gate signal remains on as $T_{\text{Gate}}$ and the beam irradiation time as $T_{IR}$.

### 2.3 Modeling delivery dynamics

For each measurement, the phantom is radiographically aligned to isocenter at the alignment phase $\varphi_{a}=25\%$. Following localization, phantom motion is initiated and the RPM system begins monitoring the surrogate. At time $t=t_{0}$ the surrogate motion reaches the specified gate‐on phase $\varphi_{0}$ and enters the gate. For a phantom executing regular periodic motion of period τ, this corresponds to $t_{0}=\left(\varphi_{0}-\varphi_{a}\right)\tau$. After some delay, at $t=t_{0}+\tau_{\mathrm{on}}$, the beam switches on and the proton spot begins to deposit a (2D) Gaussian‐shaped dose distribution on the film. Phantom motion causes the dose to smear along the direction of motion. At time $t=t_{0}+\tau_{\text{on}}+T_{\mathrm{IR}}$, the beam turns off.

Two beam‐off scenarios may occur; either the beam terminates having delivered the requested dose within a single gate‐on interval—called the Wide‐Gate case—or the beam is gated‐off because the surrogate has exited the specified gating interval—called the Narrow‐Gate case. Wide‐Gate deliveries are advantageous as they simplify the gated delivery mechanics, relying on the gating system only to initiate delivery. The feasibility of the methodology described below was initially demonstrated with a set of Wide‐Gate measurements. For Narrow‐Gate measurements, the number of monitor units (MU) was selected to ensure the delivery could not be completed within a single gate‐on interval; the delivery was manually aborted following irradiation during the first gate‐on interval. For Wide‐Gate measurements, the MU was selected such that the entire irradiation was completed within a single gate‐on interval. Each film therefore represents a single gate‐on delivery.

For both delivery scenarios, the position of the proton spot in the frame of the film is

(3) $$x\left(t\right)=A\cos\left(\omega t-\phi_{a}\right),$$

where A=1.5cm is the motion amplitude, ω=2π/τ, τ=5s is the period of the motion, and $\phi_{a}=2\pi \varphi_{a}$ is the alignment phase in radians $\left(\varphi_{a}=25\%\right)$. The average position μ of the proton spot—corresponding to the centroid of the deposited dose distribution relative to that of the BB‐shadow—is given by

(4) $$\begin{array}{ccc} \mu & = & \frac{1}{T_{\mathrm{IR}}}\int_{t_{0}+\tau_{\mathrm{on}}}^{t_{0}+\tau_{\mathrm{on}}+T_{\mathrm{IR}}}x\left(t\right)\;\mathrm{dt}\\ & = & \frac{A}{\omega T_{\mathrm{IR}}}[\sin\left(\omega \left(t_{0}+\tau_{\mathrm{on}}+T_{\mathrm{IR}}\right)-\phi_{a}\right)\\ & & -\sin\left(\omega \left(t_{0}+\tau_{\mathrm{on}}\right)-\phi_{a}\right)]. \end{array}$$

For each measurement, the beam irradiation time $\left(T_{IR}\right)$ is extracted from the accelerator log file. Inverting Equation 4 for $\tau_{on}$, we obtain

(5) $$\tau_{\mathrm{on}}=\frac{1}{\omega }\left[2\phi_{a}-\phi_{0}-\frac{\omega T_{\mathrm{IR}}}{2}-\cos^{-1}\left(\frac{\mu }{A\mathrm{sinc}\left(\frac{\omega T_{\mathrm{IR}}}{2}\right)}\right)\right].$$

Here, sinc(u)≡sin(u)/u, with sinc(0)=1. The quantity $\phi_{0}=2\pi \varphi_{0}$ is the value of the specified gate‐on phase in radians. Ideally, the BB‐shadow/spot separation, μ, is confined along the direction of motion. However, deviations in the delivered spot position and possible (slight) film rotations with respect to the phantom motion axis can induce a two‐dimensional (2D) separation. For each film, the 2D separation is extracted with a 2D binned maximum‐likelihood fit to the measured dose distribution. Both the BB‐shadow and spot signal lineshapes are modeled with 2D Gaussian probability density functions (PDFs), where the BB‐shadow PDF is negatively weighted in the fit. The lineshape used to model the background—resulting primarily from base+fog and lateral scan artifact^12^—is parameterized by a second‐order polynomial in each dimension.

Corrections are applied for both delivered spot position, reported in the accelerator log file, and a small known systematic offset between the proton treatment isocenter reported by the spot position monitor (SPM) and the x‐ray imaging isocenter, called the SPM‐calibration. This calibration is determined from a series of 14 static film measurements. For each static measurement, a piece of EBT4 film is positioned within the acrylic phantom beneath the steel BB, the BB is radiographically aligned to isocenter, and the stationary film is irradiated. Figure 3 presents the measured dose distribution and the corresponding 2D fit to extract the centroids of the BB‐shadow $\mu_{q}^{BB}$ and the spot $\mu_{q}$ (q=x,y). The (sub‐mm) systematic offset between imaging and treatment isocenters is calculated in two dimensions from static measurements as

(6) $$\delta_{q}={\left[\mu_{q}-\mu_{q}^{BB}\right]}_{\text{static}}-q_{\text{SPM}},$$

where $q_{\text{SPM}}$ is the delivered spot position measured by SPM in the proton gantry nozzle and reported in the accelerator log file. Figure 4 summarizes the extracted SPM‐calibration $\delta_{q}$. For each gated measurement, this 2D calibration is applied. For measurements of gated deliveries to moving films, the BB‐shadow/spot separation used in Equation 5 is calculated as

(7) $$\mu ={\left[\sum_{q=x,y}{\left({\left[\mu_{q}-\mu_{q}^{BB}\right]}_{\text{moving}}-q_{\text{SPM}}-\delta_{q}\right)}^{2}\right]}^{\frac{1}{2}}.$$

**FIGURE 3:**
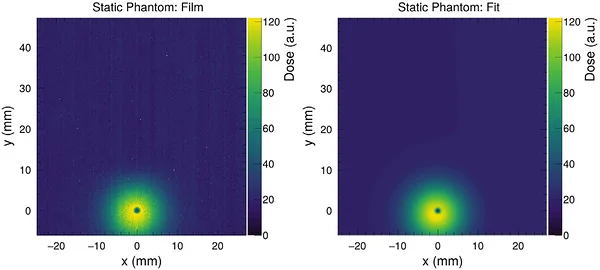
Measured dose plane (Left) and corresponding 2D‐binned fit result (Right) for a single static phantom delivery.

**FIGURE 4:**
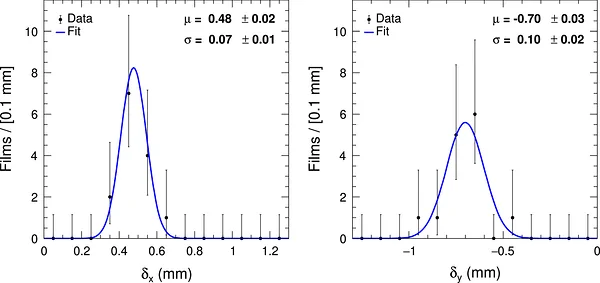
SPM‐calibrations $\delta_{x}$ (Left) and $\delta_{y}$ (Right) extracted from an unbinned fit to a set of 14 static measurements. The data are shown in 0.1 mm bins. The solid blue curve represents a Gaussian fit, and the error bars denote the Poisson statistical uncertainties in the bin counts.

Beam‐off latency is calculated for all narrow gate cases, in which the delivery is gated‐off by RPM. The total beam‐off latency is calculated according to the timeline shown in Figure 2, as

(8) $$\tau_{\text{off}}=\tau_{\text{on}}+T_{\mathrm{IR}}-\left(\varphi_{1}-\varphi_{0}\right)\tau ,$$

where $\varphi_{1}$ is the specified gate‐off phase, $\varphi_{0}$ is the specified gate‐on phase, and τ is the surrogate motion period; thus, the final term constitutes the specified gate duration. The fidelity of the log file reported values of $T_{IR}$ was verified from the oscilloscope measurements and found to be consistent within 0.2±0.1ms. The accelerator component of the beam‐on(off) latency $\tau_{\text{on}}^{\text{Acc}}\left(\tau_{\text{off}}^{\text{Acc}}\right)$ was directly measured with the oscilloscope and used to calculate the RPM‐component of the beam‐on(off) latencies using Equations 1‐2.

## 3 RESULTS

Figure 5 presents the analysis of a single Narrow‐Gate delivery, including the measured dose distribution and corresponding 2D fit result. The specified gating interval for this measurement was 25%−32%, corresponding to a gate duration of 350 ms. The phantom motion was directed rightward in the plane of the film. During delivery, the dose distribution deposited by the approximately Gaussian‐shaped proton spot smears out on the film to the left of the BB‐shadow as the phantom moves rightward. Non‐zero beam‐on/off latencies act to increase the BB‐shadow/spot separation, provided that the direction of phantom motion does not reverse during irradiation. This condition was satisfied for all measurements in this study.

**FIGURE 5:**
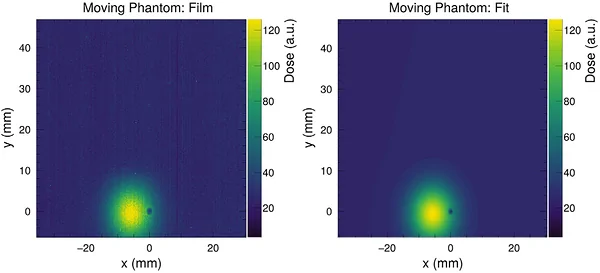
Measured dose plane (Left) and corresponding 2D‐binned fit result (Right) for a single Narrow‐Gate delivery to a moving phantom.

In total, 51 moving film measurements have been obtained: 11 Wide‐Gate and 40 Narrow‐Gate deliveries. Beam‐on latencies have been extracted from all films. Beam‐off latencies were calculated for the subset of Narrow‐Gate films, as these deliveries are gated‐off by RPM. For each measurement, the BB is radiographically aligned to isocenter at $\varphi_{a}=25\%$, but two different gate‐on phases, $\varphi_{0}=22\%\;\text{and}\;25\%$, were studied. Gate durations ranging from 3%–9% have been investigated.

Figure 6 summarizes the total beam‐on/off latency measurements. As the results are Gaussian distributed, the mean total beam‐on/off latencies for the Varian RPM + Hitachi ProBeat system are extracted from an unbinned maximum‐likelihood Gaussian fit to the data. Latency distributions are summarized as the fitted mean ± fitted standard deviation. We report a total beam‐on latency of 142±27ms for the first gate delivered in a new spill from the ProBeat synchrotron. Independent analysis of the wide‐gate data shows a similar total beam‐on latency of 171±20ms, which is consistent at the approximate 1σ‐level with the combined results. We report a total beam‐off latency of 87±30ms.

**FIGURE 6:**
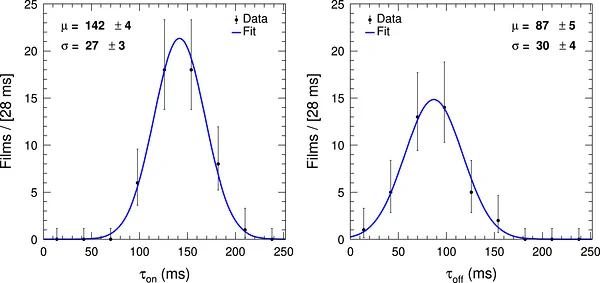
Unbinned fit to the distribution of total beam‐on (Left) and beam‐off (Right) latency measurements. The data are shown in 28 ms bins. The solid blue curve represents a Gaussian fit, and the error bars denote the Poisson statistical uncertainties in the bin counts.

Oscilloscope measurements for all 51 film deliveries demonstrated a remarkably consistent accelerator beam‐on latency of $\tau_{\text{on}}^{\text{Acc}}=69.3\pm 0.2\;\text{ms}$ for the first gate in a spill. In a separate set of 25 oscilloscope‐only measurements (with no corresponding films), the accelerator beam‐on latency for subsequent gates in the same spill and beam energy was measured as 0.53±0.07ms. We report an average accelerator beam‐off latency of 6±4ms, which has been determined from the subset of measurements where beam delivery was terminated by RPM (Narrow‐Gate cohort). To the authors' knowledge, this constitutes the first reported measurement of the beam‐on/off latencies of the Hitachi ProBeat accelerator.

Figure 7 presents the RPM‐component latencies, which are calculated by removing the accelerator contribution from the total latencies. We report average gate‐on and gate‐off latencies of 72±27ms and 81±30ms, respectively, for the RPM system. The observed gate‐on and gate‐off latencies are consistent with one another.

**FIGURE 7:**
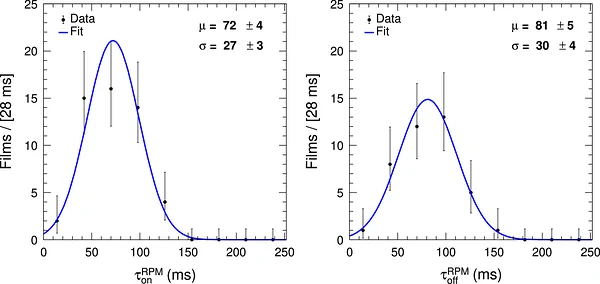
Unbinned fit to the distribution of RPM gate‐on (Left) and gate‐off (Right) latency measurements. The data are shown in 28 ms bins. The solid blue curve represents a Gaussian fit, and the error bars denote the Poisson statistical uncertainties in the bin counts.

## 4 DISCUSSION

Phase‐gated treatment assumes a reproducible relationship between respiratory phase and the target position under the regular breathing conditions represented in the treatment‐planning image set. Phase‐gating may be unsuitable in patients exhibiting persistent respiratory irregularity and should be carefully evaluated during simulation. As defined, respiratory phase is agnostic to irregularities in the underlying respiratory motion. For example, during an intermediate breath‐hold in which the patient pauses midway through exhalation, the assigned phase will continue to advance despite target position remaining approximately constant. The converse occurs during an unusually deep inspiration. In both cases, the phase error is calculable, but the corresponding target‐positional error is ambiguous and may not be clinically interpretable. This motivates clinical workflows in which phase‐gated treatments are actively monitored by an individual trained and indirectly supervised by an authorized medical physicist who manually pauses gating when irregular breathing compromises the expected relationship between respiratory phase and target position.

A variety of techniques have been reported in the literature to measure latencies associated with respiratory‐gated deliveries by photon therapy treatment systems.^9^, ^16^, ^17^, ^18^, ^19^, ^20^ Gafchromic film is by far the most widely used approach; however, to the authors' knowledge, all 2D‐based techniques reported to date infer latency from changes in the shape of dose distributions delivered to moving phantoms. While effective, analysis of measurements is time‐intensive, ultimately constraining the number of measurements that can be made in a reasonable time frame. It is possible to measure latencies without characterizing a 2D dose distribution; if the gate‐state and beam‐state are monitored concurrently with the surrogate position using a system governed by a common clock, the analysis is simplified and latency determination is expedited. Various surrogates for beam‐state have been implemented including a photon diode^19^, visible light diode^10^, and even a scintillating crystal.^21^ Mechanically coupled linear potentiometers^19^ and string/drum indexers^10^ have historically been employed to track respiratory surrogate position in real‐time. Not all clinics, however, have access to a motion phantom that provides a real‐time output signal indexing surrogate position. This is the case for our clinic; hence the motivation of this work: to develop a novel film‐based methodology for use in our proton clinic using the tools available to us. Note the same methodology could be implemented in a photon clinic with a narrow beamlet. Wiersma *et al.* ^19^ investigated several motion management systems, including a Varian Clinac equipped with a monoscopic RPM system operated in phase‐gating mode. In their study, an oscilloscope was used to simultaneously monitor RPM gate‐on/off state, surrogate position via a mechanically coupled linear potentiometer, and beam‐on/off state via a photon diode. Measurements were made for a single phase‐gating interval of 40%–60%. They report RPM gate‐on/off component latencies of 98±11ms and 90±11ms, respectively. These results are consistent with our own gate‐on/off latencies.

By comparison, the body of literature regarding latency characterization in respiratory‐gated proton therapy is limited, with even fewer studies addressing motion management systems operated in phase‐gating mode.^8^, ^10^, ^21^, ^22^, ^23^ To the authors' knowledge, the present work constitutes the first characterization of the total and constituent latencies associated with the RPM + Hitachi ProBeat system.

Recently, Worm *et al.* ^21^ characterized the latencies associated with amplitude‐gating of an RPM + Varian ProBeam (spot‐scanning cyclotron) system, using a scintillating crystal and a consumer‐grade camera manufactured by GoPro (GoPro Inc., CA, USA), reporting total beam‐on/off latencies of approximately 255 ms and 82 ms, respectively. The methodology is limited in that accelerator‐induced delays are not isolated, preventing the decomposition of the total latency into its RPM and accelerator components. Consequently, a direct quantitative comparison with the present work is not feasible.

Liu *et al.* ^10^ performed a complementary study of phase‐gated latencies for the RPM + Varian ProBeam system, with a phase‐gating interval of 40%–60%. A custom multi‐channel acquisition platform simultaneously monitored (string‐indexed) surrogate position, RPM state, and beam state (via diode reading), facilitating the extraction of constituent accelerator and RPM latencies. Total latencies, consistent with those measured by Worm *et al.*, of about 213 ms and 115 ms are reported for beam‐on/off, respectively. Accelerator constituent latencies of 108±1ms and 1.20±0.04 ms for beam‐on/off are seen for the Varian ProBeam cyclotron. The Hitachi beam‐off latency measured in the present work is of similar magnitude, with expected differences in beam‐on delays due to different accelerator dynamics. The results presented in this work complement the findings of Liu *et al.* by extending the latency characterization to a synchrotron‐based system, highlighting the importance of system‐specific gating performance evaluations in proton therapy.

The large variance (∼30ms) observed in gating latencies reported in this work is not a consequence of the precision of the methodology, but rather, is attributable to the limited sampling rate of the RPM system. The mean uncertainty on the extracted BB and spot positions is 0.17±0.06mm; propagation of positional uncertainties through the analytic simulation yields an average temporal uncertainty of 3.6 ms. The standard deviation observed in our measurements is nearly an order of magnitude larger than this. The observed dispersion in gating latencies is consistent with prior work by Lu *et al.* ^24^, who studied an RPM‐equipped (passive‐scatter) IBA synchrocyclotron and report fluctuations of ∼30ms, which they attribute to the finite sampling rate of the RPM infrared camera (30 Hz), corresponding to a temporal resolution of ∼33ms. For a fast breather, with a 20 bpm rate of respiration, this variance corresponds to ≲1% of the overall cycle.

The methodology has several limitations that should be considered when interpreting results and planning future applications. The current implementation measures beam‐on and beam‐off latencies only for the first gate in a single‐spot delivery; it does not capture the reduced latencies observed in subsequent gates within the same spill. Extending this film‐based method to multiple measurements within a single field would require a phantom with spatially separated BBs and careful consideration of spot positioning to avoid overlap on the film. For measurements of multiple gates in multi‐energy extraction mode, the interplay between energy switching and the arrival of the gating signal may introduce additional latency contributions, which would require more sophisticated techniques to characterize comprehensively.

The measurements made in this work are limited as they do not fully probe the clinically relevant phase‐gating intervals (e.g., 20%–80%). To maximize sensitivity, measurements were performed with exceptionally narrow gating intervals (3%–9%) defined near the 25% phase of the respiratory cycle. Moreover, measurements made in this work utilized a phantom executing uniform sinusoidal motion, which is a poor model for the inherent variability of patient breathing. This analysis is not sensitive to possible degradation in phase prediction fidelity in the presence of (normal) cycle‐to‐cycle variation in surrogate position. The accuracy and precision of a gating system in the presence of irregular motion (normal breathing) warrant a separate investigation, potentially with a retrospective study of patient data comparing ground truth to system‐labeled phase values.

The film‐based approach offers several advantages over alternative latency measurement techniques. Unlike electronic detection methods that rely on surrogate signals, film directly encodes latencies in the spatial distribution of the delivered dose, providing an unambiguous end‐to‐end assessment of system performance. The method is also relatively simple to implement, requiring only standard radiotherapy dosimetry equipment (Gafchromic film, flatbed scanner, analysis software) and a motion platform that is typically available in proton therapy facilities for quality assurance purposes. The spatial resolution of film (∼250μm) combined with the analytical motion model described in this work enables extraction of latency information with high precision.

The total beam‐on latency of approximately 140 ms reported in this work exceeds the 100 ms temporal accuracy threshold established by TG‐142.^7^ However, this total includes a 69 ms beam extraction judgment time that is specific to the first gate in a new synchrotron spill. For subsequent gates within the same spill, the accelerator contribution drops to the sub‐millisecond level, yielding an effective total beam‐on latency of ∼70ms, satisfying the TG‐142 recommendation.

These results underscore the importance of site‐specific latency characterization as part of gating system commissioning. We recommend measuring accelerator beam‐on and beam‐off latencies during acceptance testing and performing end‐to‐end latency measurements during commissioning. For synchrotron‐based systems, end‐to‐end testing should include the first gate in a new spill to capture any additional beam‐on delay associated with beam extraction judgement. These measurements may be supplemented with oscilloscope‐only measurements of accelerator latencies for subsequent gates within the same spill. Any significant change in RPM software version, accelerator firmware, or gating hardware should prompt a repeat of the full end‐to‐end measurement. Regular verification of gating latencies should be incorporated into quality assurance programs. The film‐based methodology presented here is well suited for commissioning‐level charact erization, where high precision is valued. For more frequent quality assurance—monthly or quarterly—one could facilitate a qualitative check by visual inspection whereby the delivered dose is centered on the BB‐shadow through careful choice of the gating interval $\left[\varphi_{0},\varphi_{1}\right)$.

For gating software with programmable offsets, where the signal could be sent early to account for latencies, it is very important that users are able to verify delivery system latencies in subsequent spills. The programmable offset should assume the minimum gate‐on latency and maximum gate‐off latency if the user wants to avoid turning on early or turning off late. The impact of an incorrect offset would be greater for shorter periods, such as for cardiac gating.

## 5 CONCLUSION

A novel, high‐precision film‐based method has been developed and validated for end‐to‐end measurement of gating latencies in spot‐scanned particle beam delivery systems, which can be generalized to x‐ray linacs. Applied to a Hitachi ProBeat synchrotron equipped with a Varian RPM gating system, the technique yields a total beam‐on latency of 142±27ms and beam‐off latency of 87±30ms for the first gate in a new spill. Decomposition of these total latencies reveals RPM‐specific contributions of 72±27ms (gate‐on) and 81±30ms (gate‐off), with accelerator‐specific contributions of 69.3±0.2ms (beam‐on, first gate in spill), 0.53±0.07ms (beam‐on, subsequent gates in existing spill), and 6±4ms (beam‐off). The observed spread in latency measurements is consistent with the temporal resolution imposed by the 30 Hz RPM camera frame rate, as the film‐based methodology does not limit the measurement precision.

Commissioning and regular quality assurance protocols to precisely characterize gating system temporal performance are essential for ensuring treatment accuracy in respiratory‐gated particle therapy. The film‐based approach presented here provides a practical, end‐to‐end validation method. As particle therapy techniques advance toward higher‐frequency gating applications—including cardiac gating and ultra‐high dose rate (FLASH) delivery systems, where temporal accuracy requirements become increasingly stringent—rigorous characterization of gating latencies will become critical. The methodology developed in this work establishes a template for such quality assurance programs and provides a benchmark for evaluating the temporal performance of motion management systems in particle therapy.

## AUTHOR CONTRIBUTIONS

All authors satisfy the four requirements outlined by the ICMJE, namely substantial contributions to the conception or design of the work; or the acquisition, analysis, or interpretation of data for the work; and drafting the work or reviewing it critically for important intellectual content; final approval of the version to be published; and agreement to be accountable for all aspects of the work in ensuring that questions related to the accuracy or integrity of any part of the work are appropriately investigated and resolved.

## CONFLICT OF INTEREST STATEMENT

The authors declare no conflicts of interest.

## ETHICS APPROVAL

This research did not involve human or animal subjects and is therefore exempt from Institutional Review Board approval.

## Data Availability

The data supporting the findings of this study are available upon reasonable request from the corresponding author.
